# Comparative Outcomes of Segmentectomy Versus Lobectomy for Solid Dominant Lung Cancer with a Tumor Diameter of 2–3 cm

**DOI:** 10.3390/jcm15062244

**Published:** 2026-03-16

**Authors:** Shota Mitsuboshi, Motoka Omata, Hiroaki Shidei, Akira Ogihara, Tamami Isaka, Masato Kanzaki

**Affiliations:** Department of Thoracic Surgery, Tokyo Women’s Medical University, Tokyo 162-8666, Japan; mitsuboshi.shota@twmu.ac.jp (S.M.); omata.motoka@twmu.ac.jp (M.O.); shidei.hiroaki@twmu.ac.jp (H.S.); ogihara.akira@twmu.ac.jp (A.O.); isaka.tamami@twmu.ac.jp (T.I.)

**Keywords:** non-small cell lung cancer, segmentectomy, lobectomy, solid-dominant, recurrence

## Abstract

**Background/Objectives**: Several studies have demonstrated feasible oncologic outcomes of segmentectomy for pure-solid or solid-dominant non-small cell lung cancer (NSCLC) measuring ≤2 cm in diameter and ground-glass opacity (GGO)-dominant NSCLC up to 3 cm in maximum tumor size. However, the applicability of segmentectomy for solid-dominant NSCLC with a tumor diameter of 2–3 cm remains controversial. This retrospective study aimed to investigate the outcomes of segmentectomy for solid-dominant NSCLC with a tumor diameter of 2–3 cm. **Methods**: We included patients who underwent lung cancer surgery at Tokyo Women’s Medical University Hospital, Tokyo, Japan, from January 2011 to December 2017. The number of patients included in this study was 743. Of the 96 eligible patients, 76 and 20 underwent lobectomy and segmentectomy, respectively. **Results**: The lobectomy and segmentectomy groups had similar 5-year overall survival rates (93.7% vs. 94.4%, respectively; HR 0.693, 95% CI 0.183–2.621, *p* = 0.586) and 5-year recurrence-free survival rates (75.8% vs. 83.6%, respectively; HR 0.639, 95% CI 0.188–2.171, *p* = 0.468). The recurrence pattern was not significantly different between the lobectomy and segmentectomy groups (locoregional 11.8% vs. 10.0%, and distant 10.5% vs. 5.0%, respectively; *p* = 0.679). Multivariable Cox regression analysis demonstrated that surgical procedure was not independently associated with OS or RFS after adjustment for confounders. **Conclusions**: Segmentectomy may be a feasible option for selected patients with solid-dominant NSCLC measuring 2–3 cm in diameter.

## 1. Introduction

For a while, the standard surgical procedure for early-stage non-small cell lung cancer (NSCLC) has been lobectomy [1]. Sublobar resection has been performed for patients with low pulmonary function or severe comorbidities. Recent randomized trials, including the JCOG0802/WJOG4607L trial and the CALGB 140503 trial, have demonstrated the oncologic feasibility of sublobar resection for selected patients with early-stage NSCLC [2,3]. Additionally, another trial has demonstrated that segmentectomy is possible even in patients with ground-glass-dominant NSCLC with tumors up to 3 cm in size [4]. Therefore, the indications for segmentectomy in early-stage NSCLC are gradually expanding. However, the applicability of segmentectomy for solid-dominant NSCLC with a tumor diameter of 2–3 cm remains controversial. Compared with ground-glass opacity (GGO)-dominant lung cancer, solid-dominant lung cancer has a higher malignant grade [5]. The disadvantages of segmentectomy compared with lobectomy need to be considered; these include the risk of locoregional recurrence due to difficulties in obtaining adequate surgical margins and efficiently assessing lymph node metastasis. In addition, while segmentectomy offers the potential benefits of parenchymal preservation and postoperative pulmonary function, concerns remain regarding oncologic safety in cases with more aggressive histologic subtypes. Recent advancements in surgical techniques and perioperative imaging have improved the precision of segmentectomy, potentially allowing for better oncologic control even in tumors larger than 2 cm. Therefore, this retrospective study aimed to investigate the outcomes of segmentectomy for solid-dominant NSCLC with a tumor diameter of 2–3 cm.

## 2. Materials and Methods

### 2.1. Study Design and Patient Population

**Figure 1:**
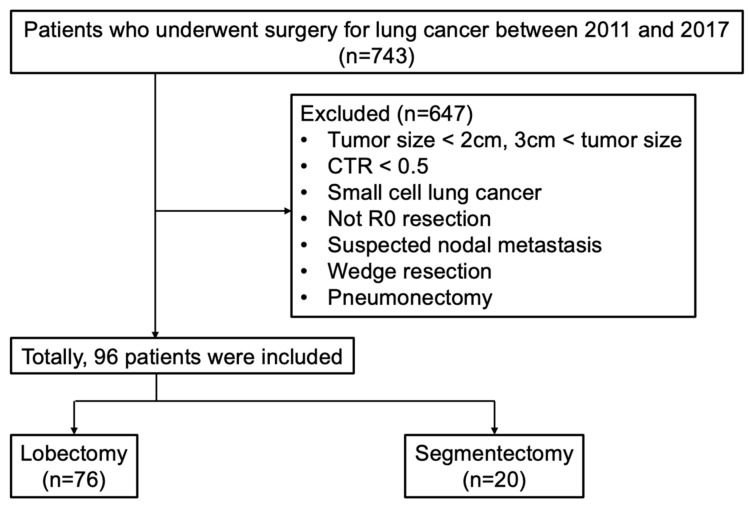
Flowchart of patient selection. In total, 96 patients were included in this study. CTR, consolidation tumor ratio.

We included patients who underwent lung cancer surgery at Tokyo Women’s Medical University Hospital, Tokyo, Japan, from January 2011 to December 2017. A total of 743 patients were screened as potential participants in this study. Patients who had tumors measuring ≥3 cm or ≤2 cm in diameter, with a consolidation tumor ratio of ≤0.5, or who had small cell lung cancer histology or suspicious lymph node metastasis; those who underwent wedge resection and pneumonectomy; and those in whom complete resection could not be achieved were excluded from this study. Ninety-six patients were included in this study; 76 and 20 underwent lobectomy and segmentectomy, respectively. The flowchart of patient selection is presented in Figure 1. This study was conducted in accordance with the Declaration of Helsinki, and the protocol was approved by the Ethics Committee of Tokyo Women’s Medical University (No. 2021-0100). Informed consent was obtained from all patients, and patient anonymity was preserved.

The patients’ clinical characteristics, computed tomography (CT) and positron emission tomography (PET) imaging, and pulmonary function were collected as preoperative evaluation data. Lymph node metastasis was determined to be negative in patients who had no swollen mediastinal or hilar lymph nodes >1 cm in short-axis diameter on high-resolution CT or in the absence of ^18^F-fluorodeoxyglucose accumulation in these lymph nodes on PET/CT. The patient characteristics, perioperative outcomes, and long-term outcomes were analyzed. This was a single-center retrospective cohort study. Data were obtained from electronic medical records, operative reports, and pathology reports.

### 2.2. Surgical Procedure

The surgical approach was either robot-assisted thoracoscopic surgery or complete video-assisted thoracoscopic surgery. For lobectomy, the pulmonary artery, veins, and bronchus were divided sequentially using endoscopic staplers or energy devices. For segmentectomy, the targeted segmental bronchus and pulmonary vessels were carefully isolated and divided. Preoperative three-dimensional (3D) pulmonary reconstruction was routinely used to plan the intersegmental plane and ensure adequate surgical margins. The margin strategy aimed to achieve a margin distance equal to or greater than tumor diameter whenever anatomically feasible. Systematic mediastinal and hilar lymph node dissection or sampling was performed according to oncological principles. In particular, patients with low pulmonary function, low performance status, and severe comorbidities underwent segmentectomy. Perioperative management, including anesthesia and postoperative care, followed institutional protocols.

### 2.3. Radiological Evaluation

The consolidation tumor ratio (CTR) was calculated as the ratio of the consolidation diameter to the maximum tumor diameter on thin-section CT. Tumors were classified as pure-solid (CTR = 1.0) or solid-dominant (CTR < 1.0).

### 2.4. Pathological Evaluation

Resected specimens were subjected to histopathological examination, and histological subtyping was conducted in accordance with the World Health Organization/International Association for the Study of Lung Cancer Histological Classification of Lung Tumors [6,7]. Tumor staging was reclassified based on the 8th edition of the tumor, node, and metastasis classification for lung cancer [8]. All specimens were fixed in 10% buffered formalin, embedded in paraffin, and stained with hematoxylin and eosin. Immunohistochemical staining was performed in cases where diagnostic clarification was required. All pathological evaluations were conducted by experienced pathologists to ensure consistency and accuracy.

### 2.5. Follow-Up

Postoperative surveillance included physical examinations, serum tumor marker assessments, and chest radiographs every 1–3 months. Additionally, chest CT scans were performed at least every 6 months, while brain magnetic resonance imaging and PET-CT were conducted when clinically indicated. Follow-up continued for at least 5 years or until death. Recurrent disease was confirmed radiologically and pathologically. Locoregional recurrence was defined as tumor recurrence in the ipsilateral thorax, including the surgical stumps in the lung or bronchus, hilar and mediastinal lymph nodes, and malignant pleural effusion. Other recurrence types were defined as distant recurrence.

### 2.6. Statistical Analysis

Continuous variables were expressed as median with interquartile range (IQR) and compared between groups using the Mann–Whitney U test. Categorical variables were expressed as numbers and percentages and compared between groups using Fisher’s exact test or the chi-square test. Survival outcomes were estimated using the Kaplan–Meier method and compared between groups using the log-rank test. To adjust for potential baseline imbalances, multivariable Cox proportional hazards regression analyses were performed. Covariates were selected based on clinical relevance and baseline imbalance between groups and included age, tumor location (left upper lobe vs. others), pure-solid proportion (CTR = 1.0 vs. <1.0), extent of lymph node dissection (ND1 vs. ND2), and postoperative adjuvant therapy. Hazard ratios (HRs) with 95% confidence intervals (CIs) were calculated. OS was calculated from the date of surgical resection to the date of death or follow-up (survival). JMP Pro software (version 17.0.0, SAS institute, Cary, NC, USA) was used for statistical analyses. A *p* value of <0.05 was considered statistically significant.

## 3. Results

### 3.1. Patient Characteristics

Table 1 shows the characteristics of the 96 patients; the median age was 68.5 years in the lobectomy group (*n* = 76) and 70.5 years in the segmentectomy group (*n* = 20). No significant differences were observed between the segmentectomy and lobectomy groups in terms of pulmonary function and comorbidities. The tumor location significantly differed between the lobectomy and segmentectomy groups. In the segmentectomy group, five (25.0%) and two (10.0%) patients underwent left upper division segmentectomy and lingulectomy, respectively.

### 3.2. Surgical Outcomes

Table 2 shows the surgical outcomes. The extent of lymph node dissection differed significantly between the lobectomy and segmentectomy groups. All patients in both groups did not require conversion to open thoracotomy and had no intraoperative complications. Although no significant difference was observed, lymph node metastasis was shown in nine (11.9%) patients who underwent lobectomy. No significant differences were observed between the two groups in terms of operation time, blood loss, pathology, and pathological stage.

### 3.3. Postoperative Outcomes

Table 3 presents the postoperative outcomes. Postoperative complications were observed in 10 (13.2%) and 2 (10.0%) patients in the lobectomy and segmentectomy groups, respectively. Persistent air leaks, atrial fibrillation, chylothorax, and pleural effusion classified as grade 2 by the Clavien–Dindo classification were observed. The 30- and 90-day mortality rates were both 0%. No patient was readmitted within 30 days. Death due to lung cancer or other causes was not significantly different between the groups. Postoperative adjuvant chemotherapy was administered to 16 patients in the lobectomy group and 3 patients in the segmentectomy group.

**Figure 2:**
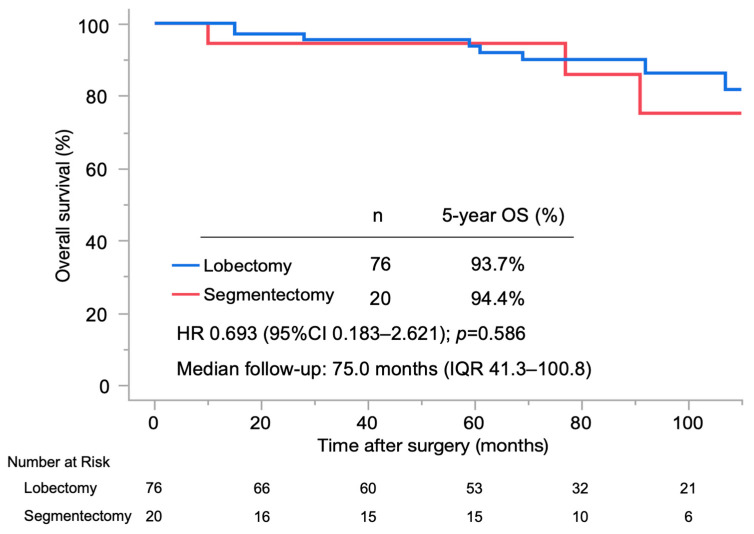
Kaplan–Meier curves of overall survival in the lobectomy and segmentectomy groups. OS, overall survival; HR, hazard ratio; CI, confidence interval; IQR, interquartile range.

**Figure 3:**
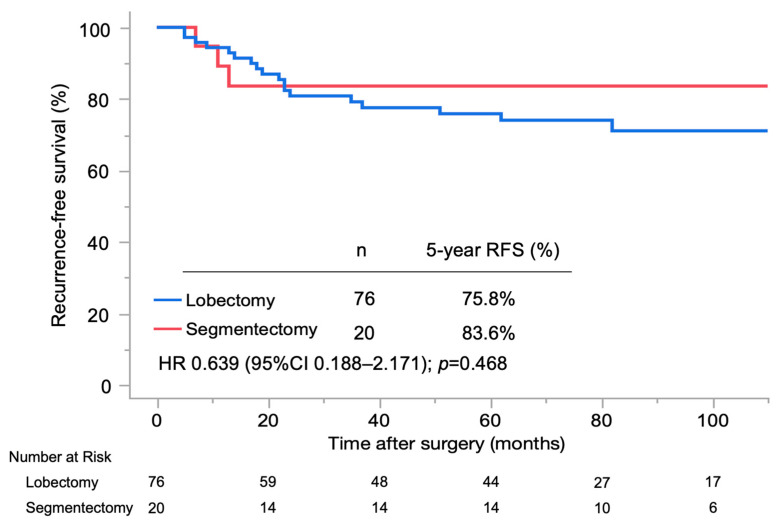
Kaplan–Meier curves of recurrence-free survival in the lobectomy and segmentectomy groups. RFS, recurrence-free survival; HR, hazard ratio; CI, confidence interval.

Figure 2 and Figure 3 show the OS and recurrence-free survival (RFS) in the lobectomy and segmentectomy groups. The median postoperative follow-up period was 75.0 months (IQR 41.3–100.8 months). The lobectomy and segmentectomy groups had a similar 5-year OS rate (93.7% vs. 94.4%, respectively; hazard ratio (HR) 0.693, 95% CI 0.183–2.621, *p* = 0.586) and 5-year RFS rate (75.8% vs. 83.6%, respectively; HR 0.639, 95% CI 0.188–2.171, *p* = 0.468). In multivariable Cox proportional hazards regression analyses adjusting for age, tumor location, pure-solid proportion, extent of lymph node dissection, and postoperative adjuvant therapy, surgical procedure was not independently associated with OS or RFS (Table 4 and Table 5).

### 3.4. Recurrence Patterns

Table 6 shows the recurrence pattern and site of the first recurrence after lobectomy and segmentectomy. Recurrence pattern was not significantly different between the lobectomy and segmentectomy groups (locoregional 11.8% vs. 10.0%, and distant 10.5% vs. 5.0%, respectively; *p* = 0.679). In the segmentectomy group, locoregional recurrence sites included a surgical stump and an ipsilateral pleural effusion in one patient (5.0%). In the lobectomy group, approximately half of the recurrence patterns were distant metastases, with sites including supraclavicular lymph node in three (3.9%), the brain in two (2.6%), and a bone, a contralateral lung, and a contralateral pleural effusion in one patient (1.3%)

## 4. Discussion

In recent years, several studies have demonstrated feasible oncologic outcomes of segmentectomy for pure-solid or solid-dominant NSCLC measuring ≤2 cm in diameter and GGO-dominant NSCLC up to 3 cm in maximum tumor size [2,4]. However, lobectomy remains the main surgical strategy for solid-dominant NSCLC with a tumor diameter of 2–3 cm. This study demonstrated that similarly to lobectomy, segmentectomy had excellent oncologic outcomes and fully acceptable locoregional control for these tumors. In the future, segmentectomy to preserve the lung is likely to be adopted as the next novel alternative to lobectomy for solid-dominant NSCLC with a tumor diameter of 2–3 cm. Segmentectomy may reduce postoperative complications in patients with low pulmonary function and various severe comorbidities.

Locoregional recurrence was more frequent after segmentectomy than after lobectomy for pure-solid or solid-dominant NSCLC measuring ≤2 cm in diameter in a previous report [2]. When deciding on the proper indications for segmentectomy for solid-dominant NSCLC with a tumor diameter of 2–3 cm, the risk of locoregional recurrence should be carefully avoided. Segmentectomy is technically more challenging than lobectomy and requires in-depth 3D knowledge of the relationship between the involved bronchi and pulmonary vessels [9,10]. We previously reported a patient-specific virtual 3D pulmonary model for thoracoscopic lung resection and applied virtual 3D pulmonary reconstruction models using homemade software, named “CTTRY” (Tokyo Women’s Medical University), for preoperative simulation [11]. These 3D pulmonary reconstruction models were used for segmentectomy to ensure sufficient identification of the surgical margin at the tumor site. Preoperative 3D simulation enabled careful planning of the resection line and assessment of the tumor-to-margin distance during segmentectomy. In the present study, this approach may have contributed to securing adequate surgical margins and achieving acceptable locoregional control. In addition, the use of patient-specific 3D pulmonary models may facilitate training and standardization of segmentectomy procedures, potentially improving safety and outcomes. In a previous report, a surgical margin of ≥2 cm was shown to prevent postoperative recurrence even in patients with pure-solid NSCLC with a tumor diameter of 2–3 cm [12]. In this study, the frequency of locoregional recurrence of solid-dominant NSCLC with a tumor diameter of 2–3 cm was similar between segmentectomy and lobectomy. Sufficient surgical margins were suggested to ensure the use of these 3D pulmonary reconstruction models for segmentectomy for solid-dominant NSCLC with a tumor diameter of 2–3 cm. However, locoregional recurrence may occur even if sufficient surgical margins are secured. Multidisciplinary therapy, including surgery, radiation therapy, chemotherapy, and immunotherapy, is important for locoregional recurrence after segmentectomy for NSCLC, which is considered to be an issue for investigation in the future. Furthermore, segmentectomy is technically challenging and may cause perioperative complications like persistent air leaks. In this study, there was no significant difference in the operation time, blood loss, or incidence of perioperative complications, and conversion to open thoracotomy and mortality were not observed. This suggested that segmentectomy was performed safely.

Compared with NSCLC with a GGO component, pure-solid NSCLC has high malignancy and higher pathologic invasiveness, including lymph node metastasis, vascular invasion, lymphatic invasion, and spread through air spaces [5,13,14]. In the present study, the median consolidation tumor ratio was 1.0 in both groups, indicating that a large proportion of tumors were pure-solid lesions. Therefore, although the inclusion criteria allowed tumors with a CTR greater than 0.5, most cases represented tumors with high malignant potential. This characteristic should be considered when interpreting the oncologic outcomes of segmentectomy in this cohort. Lymph node dissection during segmentectomy for lung cancer with a high risk of lymph node metastasis is often debated. However, no differences were found in the frequency of nodal upstaging or hilar lymph node recurrence between segmentectomy and lobectomy [2]. Moreover, post hoc analysis of that trial showed better OS after segmentectomy than after lobectomy in patients with pure-solid NSCLC [15]. Proper lymph nodal dissection for solid-dominant NSCLC with a tumor diameter of 2–3 cm is controversial. Considering the significant difference in the extent of lymph node dissection between the lobectomy and segmentectomy groups in this study, the lymph node dissection during segmentectomy might have been insufficient. This difference likely reflects differences in surgical indication and preoperative assessment rather than a true oncologic difference between procedures. Therefore, the observed imbalance in lymph node dissection extent may have introduced potential staging bias between the groups. However, this difference did not influence OS and RFS. Furthermore, the frequency of lymph node recurrence after segmentectomy was similar to that after lobectomy. Therefore, segmentectomy may be an acceptable surgical procedure even for patients at high risk of unsuspected lymph node metastasis, such as those with pure-solid NSCLC. Furthermore, lymph node metastasis from NSCLC was reported to be less frequent in peripherally located tumors than in centrally located tumors, and the risk of unsuspected lymph node metastasis in patients with peripheral pure-solid NSCLC was similar between tumors ≤ 2 cm and tumors 2–3 cm in diameter [16]. This study was unable to classify the location of the tumor as central or peripheral. Although no significant difference was observed, 11 cases of lymph node metastasis were observed in the lobectomy group and none in the segmentectomy group. Lobectomy may have been selected in centrally located tumors with a high risk of lymph node metastasis. The absence of pathological lymph node metastasis in the segmentectomy group likely reflects careful patient selection with a lower preoperative suspicion of nodal involvement. In contrast, lobectomy may have been preferentially selected for patients with tumors considered to have a higher risk of occult nodal metastasis. This difference suggests a potential baseline imbalance in oncologic risk between the groups. In addition to Kaplan–Meier survival analysis, multivariable Cox proportional hazards regression analyses were performed to adjust for potential confounding factors arising from baseline differences between the groups.

In addition, the total study population was relatively small, and the segmentectomy group consisted of only 20 patients, resulting in an imbalance between the treatment groups. Furthermore, the limited number of patients and events in the segmentectomy group may have reduced the statistical power of the study and resulted in relatively wide confidence intervals in the survival analyses. Therefore, the findings of this study should be interpreted cautiously. This imbalance reflects real-world surgical selection rather than randomized allocation, as lobectomy remained the standard procedure for solid-dominant tumors measuring 2–3 cm during the study period. Consequently, the limited number of patients and events may have reduced statistical power and resulted in relatively wide confidence intervals.

In this study, the tumor location in terms of the left and right lobes significantly differed between the lobectomy and segmentectomy groups. Half of the patients in the segmentectomy group had tumors in the left upper lobe. As dissection around the left main pulmonary artery is associated with a potential risk of intraoperative bleeding due to the diversity of pulmonary vessel branching, left upper lobe lobectomy is considered the most technically challenging procedure in lobectomy [17]. Furthermore, left upper division segmentectomy and lingulectomy were not different from left upper lobectomy for early-stage NSCLC in terms of oncological outcomes in previous reports [18]. These factors may have led to selection bias in the segmentectomy group. In addition, the higher proportion of left upper lobe tumors in the segmentectomy group may have influenced the selection of surgical procedure. Therefore, the findings of this study may not be fully generalizable to tumors located in other anatomical lobes, where surgical indications and technical considerations may differ.

Compared with lobectomy, segmentectomy can preserve postoperative pulmonary function and reduce the risk of complications. This may explain the previously reported superiority of segmentectomy over lobectomy in terms of OS among patients with pure-solid or solid-dominant NSCLC ≤ 2 cm in diameter [2]. In this study, the OS rate among patients with solid-dominant NSCLC measuring 2–3 cm in diameter was similar between segmentectomy and lobectomy. In addition, some previous studies have reported the feasibility of segmentectomy for solid-dominant NSCLC with a tumor diameter of 2–3 cm [19,20]. Compared with previous reports, the 5-year OS and RFS rates in this study were similar. Considering these results, segmentectomy could achieve acceptable survival outcomes for solid-dominant NSCLC with a tumor diameter of 2–3 cm.

The results of this study suggest the potential role of segmentectomy as a surgical option for patients with solid-dominant NSCLC measuring 2–3 cm in diameter. In patients with pulmonary dysfunction or severe complications, the ability to preserve greater amounts of lung parenchyma while achieving equivalent oncological treatment outcomes is particularly important. Furthermore, reducing the extent of resection without compromising survival outcomes may contribute to maintaining postoperative quality of life, particularly in elderly patients or those with low pulmonary function. Segmentectomy is supported as a clinically viable option not only as an alternative to lobectomy in patients unsuitable for lobectomy but also as a reasonable surgical option for those with low pulmonary function. Incorporating segmentectomy into treatment options may expand surgical indications for early-stage NSCLC and optimize personalized surgical strategies.

There are some limitations to this study. The first is the non-randomized, retrospective, and single-institution design with a small sample size, especially in the segmentectomy group. Second, this study did not evaluate postoperative patient characteristics, including surgical margin, adjuvant therapy and therapy after recurrence, which may affect survival outcomes, such as OS and RFS. Although adjuvant therapy was not analyzed, it may have influenced long-term outcomes such as OS and RFS. Third, although the median follow-up period after surgery was longer than 5 years, further follow-up is necessary to assess the long-term outcome of NSCLC segmentectomy. Fourth, there may have been selection bias in the surgical procedure due to differences in tumor location, whether central or peripheral and between the left or right lobes. It is necessary to confirm the efficacy of segmentectomy for solid-dominant NSCLC with a tumor diameter of 2–3 cm based on a prospective randomized trial. Despite these limitations, our findings suggest that segmentectomy is a feasible and oncologically acceptable option for selected patients with solid-dominant NSCLC measuring 2–3 cm. Future studies with larger, multi-center cohorts and long-term follow-up are warranted to better define the indications for segmentectomy and optimize patient selection, which could ultimately contribute to less invasive surgical strategies while maintaining comparable survival outcomes.

## 5. Conclusions

Segmentectomy may be a feasible option for selected patients with solid-dominant NSCLC measuring 2–3 cm in diameter. Further prospective randomized trials are required to confirm whether segmentectomy is appropriate for a wider range of indications.

## Figures and Tables

**Table 1:** Patients’ characteristics.

Variables		Lobectomy	Segmentectomy	*p* Value
Age, median (IQR)		68.5 (63.3–75.0)	70.5 (67.0–79.3)	0.235
Sex, *n* (%)				0.949
	Male	45 (59.2)	12 (60.0)	
	Female	31 (40.8)	8 (40.0)	
Smoking history, *n* (%)				0.375
	Never-smoker	35 (46.1)	7 (35.0)	
	Ever-smoker	41 (54.0)	13 (65.0)	
Pulmonary function				
	VC (L), median (IQR)	3.1 (2.5–3.9)	3.3 (2.5–4.1)	0.925
	%VC (%), median (IQR)	104.3 (92.2–112.6)	106.0 (84.5–133.6)	0.639
	FEV_1.0_ (L), median (IQR)	2.3 (1.8–2.9)	2.1 (1.7–2.8)	0.308
	%FEV_1.0_ (%), median (IQR)	97.7 (86.4–110.4)	89.1 (80.3–114.1)	0.384
	%DL_CO_ (%), median (IQR)	80.8 (65.8–88.6)	76.7 (59.2–92.9)	0.903
Comorbidities, *n* (%)				
	Hypertension	27 (35.5)	6 (30.0)	0.643
	Diabetes mellitus	18 (23.7)	4 (20.0)	0.727
	Other malignancy	17 (22.4)	8 (40.0)	0.110
	Cardiovascular disease	11 (14.5)	4 (20.0)	0.545
	Respiratory disease	9 (11.8)	4 (20.0)	0.343
	Cerebrovascular disease	8 (10.5)	1 (5.0)	0.451
	Renal dysfunction	4 (5.3)	1 (5.0)	0.962
	Liver dysfunction	3 (4.0)	2 (10.0)	0.278
	Collagen disease	3 (4.0)	0 (0)	0.367
	Psychiatric disease	1 (1.3)	0 (0)	0.606
cStage, *n* (%)				0.514
	IA2	12 (15.8)	2 (10.0)	
	IA3	64 (84.2)	18 (90.0)	
Tumor location, *n* (%)				0.004
	Right upper lobe	27 (35.5)	2 (10.0)	
	Right middle lobe	9 (11.8)	0 (0)	
	Right lower lobe	19 (25.0)	4 (20.0)	
	Left upper lobe	11 (14.5)	10 (50.0)	
	Left lower lobe	10 (13.2)	4 (20.0)	
Tumor size				
	Whole tumor size (mm), median (IQR)	24.4 (22–27.3)	22.4 (21.9–26.8)	0.277
	Solid component size (mm), median (IQR)	22.8 (20.1–26.7)	21.8 (20.1–23.0)	0.414
	CTR, median (IQR)	1 (0.9–1)	1 (0.8–1)	0.401
Pure-solid proportion, *n* (%)				0.176
	Pure-solid	55 (72.4)	11 (55.0)	
	Solid-dominant	21 (27.6)	9 (45.0)	

*p*-values were calculated by Fisher’s exact test or the chi-square test for categorical data and by Mann–Whitney’s U-test for continuous data. Continuous variables are expressed as the median with interquartile range, and categorical variables are expressed as numbers and percentages (%). IQR, interquartile range; VC, vital capacity; FEV_1.0_, forced expiratory volume in 1 s; %DL_CO_, percentage of diffusing capacity corrected for alveolar volume; CTR, consolidation tumor ratio.

**Table 2:** Surgical outcomes.

Variables		Lobectomy	Segmentectomy	*p* Value
Surgical approach, *n* (%)				
	Thoracoscopic surgery	76 (100)	20 (100)	
Lymph node dissection, *n* (%)				<0.001
	ND1b	16 (21.1)	15 (75.0)	
	ND2a-1	57 (75.0)	5 (25.0)	
	ND2a-2	3 (3.95)	0 (0)	
Operation time (min), median (IQR)		189 (158–226)	183.5 (144.8–197.5)	0.317
Blood loss (mL), median (IQR)		18.5 (9–58.8)	12.5 (7.8–24.3)	0.269
Conversion to open thoracotomy		0 (0)	0 (0)	
Intraoperative complications, *n* (%)		0 (0)	0 (0)	
Pathology, *n* (%)				0.635
	AD	62 (81.6)	18 (90.0)	
	SQ	13 (17.1)	2 (10.0)	
	Carcinoid	1 (1.3)	0 (0)	
Pathological LN metastasis, *n* (%)				0.271
	N1	5 (6.6)	0 (0)	
	N2	4 (5.3)	0 (0)	
pStage, *n* (%)				0.287
	IA1	6 (7.9)	1 (5.0)	
	IA2	16 (21.1)	4 (20.0)	
	IA3	29 (38.2)	13 (65.0)	
	IB	16 (21.1)	2 (10.0)	
	IIB	4 (5.3)	0 (0)	
	IIIA	5 (5.2)	0 (0)	

*p*-values were calculated by Fisher’s exact test or the chi-square test for categorical data and by Mann–Whitney’s U-test for continuous data. Continuous variables are expressed as the median with interquartile range, and categorical variables are expressed as numbers and percentages (%). ND, lymph node dissection; IQR, interquartile range; AD, adenocarcinoma; SQ, squamous cell carcinoma; LN, lymph node.

**Table 3:** Postoperative outcomes.

Variables		Lobectomy	Segmentectomy	*p* Value
Postoperative complications, *n* (%)				0.704
	Persistent air leaks	5 (6.6)	1 (5.0)	
	Af tachycardia	1 (1.3)	0 (0)	
	Chylothorax	1 (1.3)	0 (0)	
	Pleural effusion	0 (0)	1 (5.0)	
	Others	3 (4.0)	0 (0)	
Adjuvant therapy, *n* (%)		16 (21.1)	3 (15.0)	0.755
30-day mortality, *n* (%)		0 (0)	0 (0)	
90-day mortality, *n* (%)		0 (0)	0 (0)	
Death by the end of observation, *n* (%)		9 (11.8)	3 (15.0)	0.704
Cause of death, *n* (%)				0.887
	Lung cancer	4 (5.3)	1 (5.0)	
	Non-lung cancer	5 (6.6)	2 (10.0)	

*p*-values were calculated by Fisher’s exact test or the chi-square test for categorical data and by Mann–Whitney’s U-test for continuous data. Categorical variables are expressed as numbers and percentages (%). Af, atrial fibrillation.

**Table 4:** Univariate and multivariable Cox proportional hazards analyses for overall survival.

		Univariate Analysis		Multivariate Analysis	
Variables		HR (95% CI)	*p* value	HR (95% CI)	***p* Value**
Age		1.015 (0.961–1.083)	0.615	1.015 (0.951–1.091)	0.676
Procedure					
	Lobectomy (vs. segmentectomy)	0.693 (0.183–2.621)	0.589	0.762 (0.143–4.068)	0.751
Tumor location					
	LUL (vs. others)	2.601 (0.759–8.914)	0.128	2.801 (0.674–11.645)	0.157
Pure-solid proportion					
	Pure-solid (vs. solid-dominant)	1.975 (0.425–9.179)	0.385	1.899 (0.371–9.705)	0.441
Lymph node dissection					
	ND2 (vs. ND1)	0.574 (0.179–1.837)	0.350	0.668 (0.176–2.531)	0.553
Adjuvant therapy					
	Yes (vs. No)	0.747 (0.161–3.459)	0.709	0.653 (0.127–3.344)	0.609

HR, hazard ratio; CI, confidence interval; LUL, left upper lobe; ND, lymph node dissection.

**Table 5:** Univariate and multivariable Cox proportional hazards analyses for recurrence-free survival.

		Univariate Analysis		Multivariate Analysis	
Variables		HR (95% CI)	*p* value	HR (95% CI)	***p* Value**
Age		0.988 (0.951–1.030)	0.553	1.004 (0.963–1.050)	0.855
Procedure					
	Lobectomy (vs. segmentectomy)	1.565 (0.461–5.318)	0.473	1.303 (0.296–5.731)	0.726
Tumor location					
	LUL (vs. others)	1.908 (0.739–4.930)	0.182	2.396 (0.802–7.165)	0.118
Pure-solid proportion					
	Pure-solid (vs. solid-dominant)	1.745 (0.638–4.769)	0.278	1.420 (0.474–4.260)	0.531
Lymph node dissection					
	ND2 (vs. ND1)	4.327 (1.008–18.582)	0.049	4.187 (0.891–19.672)	0.070
Adjuvant therapy					
	Yes (vs. No)	2.448 (1.014–5.908)	0.046	1.870 (0.719–4.863)	0.199

HR, hazard ratio; CI, confidence interval; LUL, left upper lobe; ND, lymph node dissection.

**Table 6:** Recurrence pattern and site of the first recurrence.

Variables		Lobectomy	Segmentectomy
Recurrence, *n* (%)		17 (22.4)	3 (15.0)
Locoregional recurrence, *n* (%)		9 (11.8)	2 (10.0)
Locoregional recurrence site			
	Surgical stump	2 (2.6)	1 (5.0)
	Ipsilateral mediastinal LN	1 (1.3)	0 (0)
	Ipsilateral lung	5 (6.6)	0 (0)
	Ipsilateral pleural dissemination	1 (1.3)	0 (0)
	Ipsilateral pleural effusion	0 (0)	1 (5.0)
Distant recurrence, *n* (%)		8 (10.5)	1 (5.0)
Distant recurrence site			
	Brain	2 (2.6)	1 (5.0)
	Bone	1 (1.3)	0 (0)
	Contralateral lung	1 (1.3)	0 (0)
	Contralateral pleural effusion	1 (1.3)	0 (0)
	Supraclavicular LN	3 (3.9)	0 (0)

Categorical variables are expressed as numbers and percentages (%). LN, lymph node.

## Data Availability

All the data and materials supporting our findings are included within the article.

## Abbreviations

The following abbreviations are used in this manuscript:

NSCLC	Non-small cell lung cancer
GGO	Ground-glass opacity
OS	Overall survival
CT	Computed tomography
PET	Positron emission tomography
3D	Three-dimensional
CTR	Consolidation tumor ratio
HR	Hazard ratio
CI	Confidence interval
IQR	Interquartile range
VC	Vital capacity
FEV_1.0_	Forced expiratory volume in 1 s
%DL_CO_	Percentage of diffusing capacity corrected for alveolar volume
ND	Lymph node dissection
AD	Adenocarcinoma
SQ	Squamous cell carcinoma
LN	Lymph node
RFS	Recurrence-free survival
Af	Atrial fibrillation
